# Characteristics and Usage Patterns Among 12,151 Paid Subscribers of the Calm Meditation App: Cross-Sectional Survey

**DOI:** 10.2196/15648

**Published:** 2019-11-03

**Authors:** Jennifer Huberty, Ana-Maria Vranceanu, Colleen Carney, Michael Breus, Michael Gordon, Megan Elizabeth Puzia

**Affiliations:** 1 College of Health Solutions Arizona State University Phoenix, AZ United States; 2 Integrated Brain Health Clinical and Research Program Massachusetts General Hospital Boston, MA United States; 3 Harvard Medical School Boston, MA United States; 4 Department of Psychology Ryerson University Toronto, ON Canada; 5 Mindworks, LLC Manhattan Beach, CA United States; 6 Behavioral Research and Analytics, LLC Salt Lake City, UT United States

**Keywords:** health, psychological stress, sleep, mindfulness, meditation, consumer behavior, mobile health, mhealth, digital health

## Abstract

**Background:**

Meditation has become increasingly popular due to its health benefits; however, barriers to delivering meditation programs in traditional group-based formats limit the accessibility of these benefits. Smartphone-based meditation may increase the availability of these programs to larger, more diverse audiences; however, research on subscriber characteristics and usage patterns in meditation mobile apps is lacking.

**Objective:**

This study aimed to describe the demographics, clinical characteristics, and usage patterns of a convenience sample of Calm subscribers and explore the relationship between self-reported app usage and changes in health, stress, and sleep.

**Methods:**

Participants were 12,151 paying Calm subscribers (response rate=12.08%, 12,151/100,594) who completed an anonymous Web-based survey with 11 quantitative questions related to user engagement, reasons for starting Calm, and changes after using the app. Demographic characteristics, chronic health diagnoses, and sleep difficulties were also assessed. Chi-square tests were used to examine differences in app usage. Logistic regression models were used to examine demographic and health characteristics that may predict changes in health, stress, and sleep.

**Results:**

Respondents were 18-96 years old (mean 48.57 [SD 13.79]), primarily female (79.94%, 8778/10,981), white (81.41%, 8959/11,005), and most reported a chronic health diagnosis (56.86%, 6289/11,061). Mental health diagnoses (41.13%, 4549/11,061) were more common than physical health diagnoses (32.19%, 3560/11,061). Most respondents (76.31%, 8684/11,360) reported difficulties falling or staying asleep. On average, respondents had been using Calm for 11.49 months (SD 10.49), and 60.03% (7281/12,129) used it 5 or more times per week. Meditations (used by 80.02%, 9497/11,841) and Sleep Stories (55.66%, 6591/11,841) were the most popular components. The frequency of using Calm was associated with incremental increases in the likelihood of noticing changes in mental health (χ^2^_2_=136.8; *P*<.001), physical health (χ^2^_2_=102.8; *P*<.001), stress (χ^2^_2_=128.1; *P*<.001), and sleep (χ^2^_2_=141.4; *P*<.001). Respondents who had used Calm longer were also more likely to notice changes in mental health (OR 1.06 [95% CI 1.05 to 1.06]), physical health (OR 1.01 [95% CI 1.01 to 1.02]), stress (OR 1.04 [95% CI 1.04 to 1.05]), and sleep (OR 1.004 [95% CI 1.00 to 1.01]). Subscribers with sleep difficulties used Calm more frequently (χ^8^_2_=11.5; *P*=.003), were more likely to use Sleep Stories (χ^1^_2_=1590.2; *P*<.001), and were more likely to notice changes in their physical health (χ^1^_2_=49.2; *P*<.001) and sleep (χ^1^_2_=2391.1; *P*<.001).

**Conclusions:**

Results highlight important demographic characteristics and usage patterns among a self-selected sample of Calm subscribers. Mental health concerns and sleep appear to be top reasons for downloading Calm. Sleep Stories and meditations are the most popular app components. The frequency of using Calm was associated with incremental changes in outcomes. Findings support future randomized controlled trials testing the efficacy of Calm for health, stress, and sleep. Studies should also explore strategies to attract a more diverse sample of subscribers.

## Introduction

### Meditation Benefits

Meditation is a technique for resting the mind and attaining a state of consciousness that is different from the waking state [1]. Mindfulness meditation is the process of openly attending, with nonjudgmental awareness, to one’s present moment experience [2]. Over the last 30 years, mindfulness meditation has become increasingly popular in Western society owing to its documented mental and physical health benefits [3]. Mindfulness meditation has been linked to reductions in depressive symptoms, stress, anxiety [4,5], and improved sleep [6], through biopsychosocial mechanisms such as alterations in brain structure, improved attention, increased emotional regulation, and better immune function [4]. Given these benefits, mindfulness meditation has been incorporated routinely in psychotherapy, school-based programs, corporate settings, prisons, and military and is an important part of hospital preventative or disease management programs [3].

### Meditation Accessibility

Although the evidence for the use of mindfulness meditation is slowly growing, there are multiple barriers to its delivery in traditional group-based formats [7], thus limiting its reach and its potential to be used by individuals seeking to manage mood, stress, or symptoms of a chronic illness. First, participation requires people to travel to a public location with a trained meditation instructor at a specific time of day [5]. This is often not feasible for individuals with diagnoses such as depression, anxiety, or posttraumatic stress for whom attrition rates are as high as 50% [7,8]. Caregivers, parents, and individuals with multiple responsibilities and high stress have busy schedules and may not be able to prioritize lengthy (eg, 60-90 min) weekly classes for extended period [7]. Individuals who live in rural areas or those with chronic disease may find travel to clinics for meditation sessions burdensome [9-11]. To overcome these barriers to participation, we need novel methods to deliver mindfulness meditation.

#### Meditation and Smartphone Apps

Smartphone apps may be an effective way to deliver mindfulness meditation to geographically diverse and dispersed individuals that bypasses barriers associated with the traditional format of mindfulness meditation programs [5]. Meditation delivered on a smartphone app may retain the elements of traditional meditation programs (ie, a trained instructor delivering high-quality meditation), while allowing participants to tailor the type and length of meditations, use behavioral components to increase adherence (eg, trackers, reminders, and social support communities), and choose when and where to engage with the app. Furthermore, there is relatively less cost associated with meditation delivered on an app. Approximately 81% of US adults report having a smartphone [12] and, around the world, smartphone usage is rapidly increasing [13]. In a cross-sectional survey of 1604 mobile phone users in the United States, 58% had downloaded a health-related mobile app that they used daily.

Despite their promise, the growing popularity of mindfulness-based apps, and efforts that are currently being made to evaluate and improve the science behind these apps, knowledge gaps remain [14]. To date, efficacy studies have had small samples, inadequate control groups, and short follow-up periods [5,15]. More importantly, data related to usage patterns of mindfulness meditation apps, demographics, clinical characteristics of users, reasons for downloading health apps, barriers to use, and consistency of use are lacking [16]. Furthermore, we do not know which personal characteristics predispose one to benefit more from specific app content [14]; this information is pivotal to building content and determining the app’s effects. Unfortunately, app developers do not publish information on their users or the extent to which consumers continue to use apps over time [14]. When reports are available to nonindustry audiences, they are accompanied by fees, making them less accessible to the general public and researchers.

### Study Aims

The aim of this study was 2-fold. First, we reported demographics, clinical characteristics, and usage patterns in a self-selected sample of paying Calm subscribers. Second, we explored the relationship between the self-reported frequency of Calm use and the app’s individual components with self-reported changes in health (mental and physical), stress, and sleep. We hypothesized that subscribers who use Calm more frequently will be more likely to report changes in their health, stress, and sleep.

## Methods

### Ethics Approval

The Institutional Review Board at Arizona State University (STUDY00009725) approved the study. All participants provided electronic consent before participating in the survey. The datasets generated or analyzed during the study are available from the corresponding author upon request.

### Study Design/Recruitment

This study was cross-sectional. Participants were paying subscribers to the mobile app, Calm (see Multimedia Appendix 1 for screenshots). We used the mobile app, Calm, owing to its popularity. According to a 2015 systematic review of mindfulness apps, there are a total of 700 apps in Apple and Google Play app stores [17]. Calm was the number 1 downloaded health and fitness app between June 2018 and 2019 [18]. In an August 2019 review, CNET (a top website for technology news and editorials) confirmed that Calm was among the most highly rated meditation apps in the Apple and Google Play app stores (all above 4 stars) [19].

Calm guides users through mindfulness meditation. The 7 Days of Calm (ie, introductory course) introduces the user to mindfulness meditation and provides education about beginning a meditation practice. Users receive a new 10-min daily meditation (ie, *The Daily Calm*) and have access to a variety of meditation content (eg, reducing anxiety and depression, increasing compassion and gratitude) that ranges from 3 to 35 min. Calm offers a variety of meditations for sleep similar to other mindfulness-based apps [20]; however, unlike other apps, Calm offers Sleep Stories. Sleep Stories are narrated fictional stories using traditional storytelling methods, helping to immerse the user in their senses to improve sleep. Other content that Calm offers includes Calm Breathe (breathing training exercises), Calm Music (relaxing, soothing music and nature sounds), Calm Masterclass (monthly series of in-depth audio classes, delivered by experts that teach skills to deal with common stressors and improve well-being), and Calm Body (video lessons on slow, mindful movement routines). Calm also offers a variety of theory-based behavioral strategies to help the user be successful at regular participation including reminders to meditate, ability to track time spent meditating, and opportunities to share progress on social media.

Participants were recruited in April 2019. Calm subscribers were eligible if they were at least 18 years of age, their subscription expiration was at least two months away, they had opened at least two emails from Calm in the last 30 days, and they had completed at least two Calm sessions in the last 30 days.

Subscribers received an email inviting them to answer a series of quantitative and qualitative questions related to their usage of Calm and were informed that their answers would help improve their experience with the app, and results of this study may be used in reports, presentations, or publications. Approximately, 4744 subscribers were contacted on April 9, 54,193 were contacted on April 10, and 41,657 were contacted on April 11. Of the 100,594 subscribers who were contacted, 12,151 (12.08%, 12,151/100,594) participated in the survey. At the end of the survey, participants had the option of providing an email address to be contacted for future studies or to be entered into a draw to win 1 of 2 US $99 Amazon gift cards. No other identifying information was collected, and responses were not linked to in-app usage data.

### Survey

The survey was developed by 3 doctoral-level researchers/clinicians in the field of behavior change, complementary approaches, and sleep. The survey was Web-based (delivered using Qualtrics) and took most participants between 5 and 10 min to complete (median=6.9; interquartile range=5.7). All responses were anonymous. Participants completed 11 multiple choice quantitative questions (not including demographics) about (1) their engagement with the Calm app (4 questions), (2) why they started using Calm (1 question), (3) clinical characteristics at the time they downloaded the app (2 questions), (4) their connections with other Calm users (3 questions), and (5) whether they noticed changes in their mental health, physical health, stress, or sleep after using Calm (1 yes or no question, separated into 4 subquestions about mental and physical health, stress, and sleep; Textbox 1). Questions varied slightly based on answers to frequency of use (see Multimedia Appendix 2 for all quantitative/multiple choice questions). At the end of the survey, participants completed 4 questions about demographic characteristics. Although the survey included 3 additional open-ended questions, the scope of this paper was limited to discussion of findings from the quantitative data; qualitative analyses will be included in future publications.

**App usage questions from the Calm user survey.**

Question:

1. How often do you use the Calm app?

2. Why did you start using the Calm app?

3. When you downloaded Calm, had you been diagnosed with any of the following chronic conditions? (eg, anxiety and high blood pressure)

4a. Which components of the app do you use most often? (eg, meditations and Sleep Stories)

4b. (For selected components only) How often do you use (component) on the Calm app?

5. When you first started using Calm, did you have difficulty falling asleep or staying asleep?

6. Have you used any of the following to help increase how frequently you use the app? (eg, reminders and Calm Facebook community)

7. For how many months have you been using Calm?

8. Have you noticed any changes in your (sleep/stress/mental health/physical health) after using Calm?

9. Research shows that it is easier to use an app if friends and family are also using the same app and can share their progress with each other. Do any of your friends and/or family members use Calm?

10. (If friends and/or family members use Calm) Do you communicate with your friends/family about using the Calm app?

11. Would you want to be connected with other app users?

### Statistical Analysis

Data were analyzed using IBM SPSS 25.0. All variables were categorical, with the exception of age. For questions about health diagnoses, sleep difficulties, reasons for starting Calm, the components of Calm used, and tools used to support engagement, participants were able to endorse multiple items from a list of options; selected options were treated as endorsements (eg, used meditations), and unselected options were treated as nonendorsements (eg, did not use meditations). As not all participants answered every question, sample sizes differed across analyses. Usage frequency was categorized as ordinal, reflecting use of the app or component of the app as nonendorsed, 1 to 2 times per week, 3 to 4 times per week, or 5 or more times per week. Variables for noticing changes in health, stress, and sleep were dichotomous.

Chi-square tests were used to evaluate differences in the use of tools for engagement and frequency of using Calm. To examine the relationships between demographic characteristics and the frequency of using Calm and its components, and we used chi-square tests (sex differences) and binomial logistic regressions (age). Differences in the frequency of using Sleep Stories were further explored using ordinal logistic regressions controlling for sleep difficulties. Chi-square tests were used to analyze differences in the frequency of using Calm and the components of Calm used based on health diagnoses and sleep difficulties. We also used chi-square tests to investigate the relationship between the frequency of using Calm and noticing changes in health, stress, sleep and the relationship between sleep difficulties and noticing changes in health, stress, and sleep. In all cases, significant chi-square tests were followed up with *z* tests of column proportions. *P* values were adjusted for multiple comparisons using the Bonferroni correction.

## Results

### Demographic Characteristics

A total of 12,151 subscribers participated in the survey. Sample demographics are presented in Table 1.

**Table 1:** Demographics of Calm subscribers (N=12,151).

Category			Values, n (%)
**Age (years; N=11,010; mean** **48.6 [SD 14.0])**			
	18-24	279 (2.53)	
	25-44	4230 (38.42)	
	45-64	4904 (44.54)	
	≥65	1597 (14.50)	
**Gender (N=10,981)**			
	Female	8778 (79.94)	
	Male	2177 (19.83)	
	Other	26 (0.24)	
**Race (N=11,005)**			
	White, European American, or Caucasian	8959 (81.41)	
	Asian or Asian American	413 (3.75)	
	Black, African American, or Native African	153 (1.39)	
	Other	1480 (13.45)	
**Annual income (US $; N=9474)**			
	<21,000	665 (7.02)	
	21,000-60,000	2536 (26.77)	
	61,000-100,000	2539 (26.80)	
	>100,000	3734 (39.41)	

### Clinical Characteristics

More than half (56.86%, 6289/11,061) of respondents had a chronic health diagnosis. Mental health diagnoses (ie, depression, anxiety, or posttraumatic stress disorder; 41.13%, 4549/11,061) were more common than physical health diagnoses (ie, high blood pressure, pain, high cholesterol, asthma, arthritis, cancer, heart disease, emphysema or chronic obstructive pulmonary disease, or other lung disease; 32.19%, 3560/11,061). The most commonly reported mental health diagnosis was anxiety (33.22%, 3675/11,061), and the most common physical health diagnosis was high blood pressure (12.68%, 1402/11,061; Table 2).

Most respondents (76.49%, 8704/11,380) reported sleep difficulties (ie, difficultly falling or staying asleep; Table 3), and 13.90% (1537/11,061) reported insomnia diagnoses (Table 2). Sleep difficulties were significantly more common in females (80.09%, 7009/8751) than in males (61.77%, 1336/2163; *χ^2^_1_*=355.9; *P*<.001).

**Table 2:** Self-reported mental and physical health diagnoses in Calm subscribers (N=11,061).

Diagnosis	Values, n (%)
Anxiety	3675 (33.22)
Depression	2720 (24.59)
Insomnia	1537 (13.90)
High blood pressure	1402 (12.68)
Pain	1006 (9.10)
High cholesterol	905 (8.18)
Asthma	773 (6.99)
Posttraumatic stress disorder	772 (6.98)
Arthritis	698 (6.31)
Diabetes	330 (2.98)
Cancer	265 (2.40)
Heart	206 (1.86)
Emphysema or chronic obstructive pulmonary disease	50 (0.45)
Other lung disease	49 (0.44)
Other chronic condition	1076 (9.73)
None	4772 (43.14)

**Table 3:** Self-reported sleep difficulties in Calm subscribers (N=11,380).

Type of sleep difficulty	Values, n (%)
Difficulty falling asleep only	2664 (23.41)
Difficulty staying asleep only	2112 (18.56)
Difficulty both falling and staying asleep	3928 (34.52)
No difficulty falling or staying asleep	2676 (23.51)

### Usage Patterns

The most common reasons for starting to use Calm were to improve sleep (62.97%, 7475/11,870) and to improve stress (62.11%, 7373/11,870), followed by reducing depression or anxiety (54.47%, 6465/11,870) and improving overall health (40.05%, 4754/11,870; see Multimedia Appendix 3 for less frequently reported reasons).

#### Overall Usage of Calm and Its Components

Respondents had been using Calm for an average of 11.49 months (SD 10.49), with 55.89% (6042/10,810) having used the app for less than 1 year. Most of them used Calm 5 or more times per week (60.03%, 7281/12,129), with 27.45% (3330/12,129) using 3 to 4 times per week and 12.52% (1518/12,129) using 1 to 2 times per week. Meditations were the most popular components (80.20%, 9497/11,841), followed by Sleep Stories (55.66%, 6591/11,841; see Multimedia Appendix 4 for usage of less popular components).

Approximately, half (51.09%, 6111/11,962) of respondents used at least one tool to help, motivate, or encourage themselves to use Calm. Tracking (36.62%, 4380/11,962) and reminders (15.81%, 1891/11,962) were the most common tools. The least common tools were the Calm Facebook community (3.33%, 398/11,962) and sharing app usage on social media (2.40%, 287/11,962).

Low-frequency users (ie, using Calm 1 or 2 times per week) were significantly more likely to use the reminders (*χ^2^_1_*=84.2; *P*<.001), whereas high-frequency users (ie, using Calm at least three times per week) were more likely to use meditation tracking (*χ^2^_1_*=56.2; *P*<.001) and the Calm Facebook community (*χ^2^_1_*=7.5; *P*=.01). When asked what encouraged or motivated high-frequency users to use the app, 42.34% (4493/10,611) reported that they used Calm before bed, and 53.33% (5659/10,611) were *committed* to using Calm.

A total of 40% (4500/11,249) of respondents had friends or family members that also used Calm, and, of those, 75.00% (3375/4500) talked with their friends or family about using the app. However, when participants in the overall sample were asked whether they would like to be connected with other Calm users, 81.59% (9178/11,249) declined. Respondents with mental health diagnoses were significantly more likely to want to connect with other Calm users (22.37%, 961/4295) than those without mental health diagnoses (15.24%, 1051/6895; *χ^2^_1_*=91.3; *P*<.001). Desire to connect with other Calm users was not related to physical health diagnoses (18.35%, 622/3390 vs 17.82%, 1390/7800 without; *χ^2^_1_*=0.4; *P*=.50).

#### Differences in Usage by Demographics

There were no significant gender differences in how often respondents used Calm (*χ^2^_2_*=4.3; *P*=.12). Males were significantly more likely to use meditations (88.38%, 1924/2177) than females (77.87%, 6835/8778; *χ^2^_1_*=120.3; *P*<.001), whereas females (60.42%, 5304/8778) were more likely to use Sleep Stories than males (35.42%, 771/2177; *χ^2^_1_*=441.6; *P*<.001). Females also used Sleep Stories more frequently, even when controlling for sleep difficulties (OR 0.56 [95% CI 0.49 to 0.63]; *P*<.001). Older users tended to use the app more frequently (OR 1.02 [95% CI 1.01 to 1.02]) and were more likely to use Sleep Stories (OR 1.01 [95% CI 1.0106 to 1.011]); younger respondents were more likely to use meditations (OR 0.99 [95% CI 0.98 to 0.99]).

#### Calm Usage Based on Health Diagnoses

Participants were separated into 4 groups based on diagnoses when they downloaded Calm: (1) mental health diagnoses only (ie, no comorbid physical health diagnoses), (2) physical health diagnoses only (ie, no comorbid mental health diagnoses), (3) both physical and mental health diagnoses, and (4) no chronic health diagnoses (Table 4).

Respondents with physical health diagnoses only or with both mental and physical health diagnoses used Calm more frequently (ie, 5 or more times per week) than respondents with only mental health diagnoses or with no chronic health diagnoses (*χ^2^_6_*=77.3; *P*<.001; Table 5). Respondents with only mental health diagnoses were most likely to use meditations, followed by those with no chronic health diagnoses (*χ^2^_3_*=38.9; *P*<.001); conversely, those with no chronic health diagnoses were least likely to use Sleep Stories, whereas those with physical health and mental health diagnoses were most likely to use Sleep Stories (*χ^2^_3_*=52.0; *P*<.001).

Subscribers with sleep difficulties used Calm more frequently (ie, 5 or more times per week; *χ^2^_8_*=11.5; *P*=.003) and were significantly more likely to use Sleep Stories (*χ^2^_1_*=1590.2; *P*<.001; Table 6). Those without sleep difficulties were more likely to use meditations (*χ^2^_1_*=273.2; *P*<.001).

**Table 4:** Types of self-reported health diagnoses in Calm subscribers (N=11,061).

Diagnosis type	Values, n (%)
Mental health^a^ only	2729 (24.67)
Physical health^b^ only	1740 (15.73)
Mental and physical health	1820 (16.45)
No chronic health diagnoses	4772 (43.14)

^a^Mental health diagnoses were depression, anxiety, and posttraumatic stress disorder.

^b^Physical health diagnoses were high blood pressure, pain, high cholesterol, asthma, arthritis, cancer, heart disease, emphysema or chronic obstructive pulmonary disease, and other lung disease.

**Table 5:** Differences in the frequency of using Calm based on health diagnoses (N=11,061).

Category		Only mental health diagnoses (N=2729), n (%)	Only physical health diagnoses (N=1740), n (%)	Mental and physical health diagnoses (N=1820), n (%)	No chronic health diagnoses (N=4772), n (%)
**Frequency of using Calm (times per week)**					
	1-2^a^	324 (11.87)	172 (9.89)	200 (10.99)	609 (12.76)
	3-4^a^	778 (28.51)	411 (23.6)	440 (24.18)	1407 (29.48)
	5+^a^	1627 (59.62)	1157 (66.49)	1180 (64.84)	2756 (57.75)
**Components used**					
	Meditations^a^	2279 (83.51)	1325 (76.15)	1434 (78.79)	3808 (79.80)
	Sleep Stories^a^	1515 (55.51)	1021 (58.68)	1116 (61.32)	2495 (52.28)

^a^Chi-square was statistically significant; *P*<.05.

**Table 6:** Differences in the frequency of using Calm, frequency of using meditations and Sleep Stories, and reasons for starting to use Calm based on reported sleep difficulties (N=11,360).

Category		Sleep difficulties (N=8684), n (%)	No sleep difficulties (N=2676),n (%)
**Frequency of using Calm (times per week)**			
	1-2	1020 (11.75)	367 (13.71)
	3-4	2323 (26.75)	748 (27.95)
	5+^a^	5361 (61.73)	1561 (58.33)
**Components used**			
	Meditations^a^	6677 (76.89)	2431 (90.84)
	Sleep stories^a^	5589 (64.36)	755 (28.21)

^a^Chi-square test was statistically significant; *P*<.05.

#### Relationship Between Frequency of Calm Use and Noticing Changes in Health, Stress, and Sleep

The frequency of using Calm was associated with incremental increases in the likelihood of noticing changes in mental health (*χ^2^_2_*=136.8; *P*<.001), physical health (*χ^2^_2_*=102.8; *P*<.001), stress (*χ^2^_2_*=128.1; *P*<.001), and sleep (*χ^2^_2_*=141.4; *P*<.001; Table 7). In addition, participants who had been using Calm longer were more likely to notice changes in mental health (OR 1.06 [95% CI 1.05 to 1.06]; *P*<.001), physical health (OR 1.01 [95% CI 1.01 to 1.02]; *P*<.001), stress (OR 1.04 [95% CI 1.04 to 1.05]; *P*<.001), and sleep (OR 1.004 [95% CI 1.00 to 1.01]; *P*=.03).

**Table 7:** Differences in noticing changes in mental health, physical health, stress, and sleep based on frequency of using Calm.

Reported changes		Did not use regularly, n (%)	Used 1-2 times per week, n (%)	Used 3-4 times per week, n (%)	Used 5+ times per week, n (%)
**Calm (N=12,192)**		—^a^	1518 (12.97)	3330 (27.31)	7281 (59.72)
	Mental health^b^	—	932 (72.08)	2325 (76.56)	5734 (83.83)
	Physical health^b^	—	594 (46.19)	1558 (51.35)	4045 (59.13)
	Stress^b^	—	875 (68.15)	2299 (75.97)	5550 (81.46)
	Sleep^b^	—	745 (58.16)	2011 (66.02)	5028 (73.27)
**Meditations (N=11,189)**		2248 (20.09)	1889 (16.88)	2832 (25.31)	4220 (37.72)
	Mental health^b^	1209 (54.56)	1490 (78.46)	2430 (85.96)	3862 (91.34)
	Physical health^b^	1053 (47.14)	973 (51.51)	1598 (56.47)	2573 (61.15)
	Stress^b^	1192 (53.94)	1428 (75.76)	2386 (84.61)	3718 (88.36)
	Sleep^b^	1890 (84.07)	1304 (69.03)	1812 (63.98)	2778 (65.83)
**Sleep Stories (N=11,189)**		4959 (44.32)	2103 (18.80)	1866 (16.68)	2261 (20.21)
	Mental health^b^	4272 (85.78)	1696 (80.46)	1381 (74.69)	1642 (73.53)
	Physical health^b^	2721 (54.84)	1113 (52.92)	1016 (54.65)	1347 (60.21)
	Stress^b^	4181 (84.24)	1607 (77.00)	1341 (72.53)	1595 (71.72)
	Sleep^b^	2551 (50.84)	1545 (73.47)	1641 (87.94)	2077 (91.86)

^a^Cells containing — indicate that the response option in that column was not applicable to that question.

^b^Chi-square test was statistically significant; *P*<.05.

Frequency of using meditations was associated with significant, incremental increases in the percentage of respondents who noticed changes in their physical health (*χ^2^_3_*=130.9; *P*<.001), mental health (*χ^2^_3_*=1325.0; *P*<.001), and stress (*χ^2^_3_*=1100.5; *P*<.001; Table 7). The frequency of using meditation was not associated with changes in sleep (OR 1.04 [95% CI 0.99 to 1.01]; *P*=.12) after controlling for sleep difficulties.

Frequency of using Sleep Stories was associated with significant, incremental increases in the percentage of respondents who noticed changes in their sleep (*χ^2^_3_*=1665.3; *P*<.001; Table 7), and respondents who used Sleep Stories 5 or more times per week were significantly more likely to notice changes in physical health (*χ^2^_3_*=27.2; *P*<.001). However, there were significant negative associations between the frequency of using Sleep Stories and noticing changes in mental health (*χ^2^_3_*=197.3; *P*<.001) and stress (*χ^2^_3_*=199.0; *P*<.001). Specifically, Sleep Stories users were less likely to notice changes in their mental health and stress, and low-frequency users (ie, 1-2 times per week) were more likely to notice changes in mental health and stress than more frequent users; see Multimedia Appendix 5 for changes associated with other Calm components.

#### Relationship Between Sleep Difficulties and Noticing Changes in Health, Stress, and Sleep

Respondents with sleep difficulties were more likely to notice changes in their physical health (*χ^2^_1_*=49.2; *P*<.001) and sleep (*χ^2^_1_*=2391.1; *P*<.001) after using Calm (Table 8), whereas those without sleep difficulties were more likely to notice changes in their mental health (*χ^2^_1_*=33.1; *P*<.001) and stress (*χ^2^_1_*=17.0; *P*<.001).

**Table 8:** Differences in noticing changes in mental health, physical health, stress, and sleep based on reported sleep difficulties (N=11,131).

Reported changes	Sleep difficulties (N=8527), n (%)	No sleep difficulties (N=2604)*,* n (%)
Mental health^a^	6755 (79.30)	2212 (84.40)
Physical health^a^	4890 (57.35)	1290 (49.54)
Stress^a^	6576 (77.57)	2126 (81.36)
Sleep^a^	6961 (81.35)	808 (31.02)

^a^Chi-square test was statistically significant; *P*<.05.

## Discussion

### Summary

The aim of this paper was to report demographic characteristics, clinical characteristics, and usage patterns in a self-selected sample of paying subscribers of a consumer-based mobile app (ie, Calm). In addition, we explored the relationship between self-reported frequency of using Calm and its components and noticing changes in health (mental and physical), stress, and sleep disturbance. This is the first paper to report the characteristics of Calm subscribers in addition to the relationship between Calm use and reported changes in health, stress, and sleep.

Respondents were mostly white and mostly female. More than half reported a mental or physical health diagnosis, and approximately three-fourth reported sleep difficulties. The most common reason for starting to use Calm was for sleep, followed by stress and depression. Most respondents used Calm 5 or more times per week.

Meditations and Sleep Stories were the most commonly used components. It is not surprising that meditation was the most frequently used Calm component as Americans are more consistently including meditation as part of their lifestyle [21]. Meditation is the fifth most common type of complementary approach to health practiced by adults [22]. In addition, Calm was originally marketed as a mindfulness meditation app, and after many iterations and changes to the app over time, it now offers added components such as Sleep Stories.

### Gender Differences

Even though our sample was mostly women, men were slightly more likely to use meditation. Historically, women report higher use of meditation when compared with men. In a 2019 analysis of 2012 data from the National Health Interview Survey [21], 5.7 million men reported using meditation as compared with 12.2 million women. Both men and women reported meditation being helpful to reduce stress. In future studies, it would be interesting to know why men use the meditations more than the many other components of the app. This information may impact the way in which consumer-based meditation apps consider marketing content based on gender. For example, advertising Calm with pictures of men meditating may help to encourage men to subscribe to the app.

Women were more likely than men to use the Sleep Stories. This may be partially attributed to women reporting higher levels of sleep difficulties. It is well known that women have a higher frequency of sleep disturbance as compared with men owing to hormonal changes over the life span [23]. Sleep meditations may represent a manageable, nonpharmacological strategy for women (and men) to improve sleep. However, further analyses suggest that sleep difficulties do not fully account for women’s more frequent use of Sleep Stories. Future research using surveys and qualitative data from consumers (both men and women) about what they specifically enjoy about the Sleep Stories would be useful information for future content development, consumer engagement, and clinical trials.

### Tools for Engagement

Tracking and reminders were the most common in-app tools used to support engagement, and using the app at bedtime (using Calm at bedtime) appeared to be the most effective adherence strategy to maintain engagement with the app over time. Research on the efficacy of mobile apps in impacting behavior shows that tracking behavior and feedback are among the most successful strategies for behavior change [24]. Tracking is also one of the most popular technology strategies used by mobile apps for health and wellness [25]. Almost 40% of our sample reported using tracking as a tool to help them continue to engage in using Calm. Currently, Calm tracks how many sessions and minutes the user has participated in over time. Recent evidence suggests that tracking plus feedback may be more impactful for improvements in behavior change as compared with tracking alone [26-28]. For example, in a randomized clinical trial testing the effects of an automated tracking-texting intervention on physical activity (mActive), coupling text messages containing automated personalized feedback with physical activity tracking led to twice as many participants achieving their daily step goal as compared with other groups [26]. Providing the subscriber feedback, based on the use of the app, could thus be an effective strategy for consumer-based mindfulness meditation apps to keep subscribers engaged once they sign up as a paying member. In addition, finding other ways to track and provide feedback outside of sessions and minutes of meditation could provide more value to a meditation app. For example, the users’ perception of their stress, mindfulness, or happiness after a session could be tracked in conjunction with patterns of use. Providing this information as feedback would allow users to observe the relationship between using Calm and changes in their moods, thoughts, and behaviors. Future research in this area is warranted.

When we asked those who engaged in the app more often (more than 3 times per week) precisely how they stay engaged, many reported that using Calm at bedtime (eg, Sleep Stories, Daily Calm, and other sleep meditations) contributed to adherence. It is well known that there are barriers to beginning and maintaining health behaviors (eg, physical activity, diet, and meditation) [29]. However, using Calm (and other mindfulness-based apps) at bedtime/for sleep may have fewer barriers (eg, time and motivation) than participating in another health behavior. For example, Calm Sleep Stories require the user to begin the Sleep Story by lying in bed and assuming a comfortable position with the goal of helping the user to fall asleep. This does not require added time or motivation (beyond starting the Sleep Story) and may be an easier task, thus users reported using Calm at bedtime/for sleep as a way of using the app more consistently.

An interesting finding from our survey was that almost half of our sample had friends or family that used Calm, and many talked with their friends or family about Calm. However, overall, respondents reported not wanting to be connected with other Calm users. When exploring mental or physical health diagnoses and the relationship to reporting the desire to be connected to other Calm users, those with mental health diagnoses wanted to connect with other Calm users. Recent research suggests that individuals with mental health problems are increasingly turning to online communities for social support [30,31]. Although more than half of the survey sample started using Calm for mental health reasons (ie, anxiety and depression), and almost half reported a mental health diagnoses (ie, anxiety, depression, and posttraumatic stress); only 3% used the Calm Facebook community to maintain app engagement. This is surprising given the strong presence of Calm on Facebook and other social media platforms. It may be that this convenience sample of highly engaged users already felt well connected in terms of social support, and thus, their feedback on additional support might not be generalizable. It is also possible that concerns related to privacy and stigma deter users with mental health diagnoses from disclosing personal or emotional information to a larger community [32]. These individuals may prefer to connect specifically with other users who share common difficulties [33,34]. Future investigation is necessary to determine what the best way is to connect users and to facilitate social support for subscribers of these apps.

### Changes in Health, Stress, and Sleep

Participants using Calm more frequently were incrementally more likely to notice changes in both mental and physical health, stress, and sleep, as were those who reported using Calm for a longer duration. This is not surprising. Participants are likely becoming more mindful and reap the benefits associated with mindfulness [35,36]. Present literature on the amount of meditation practice needed to observe changes in a range of health outcomes is varied and limited [37]. However, there is promising evidence for greater effects on health, stress, and sleep as time spent (eg duration, frequency) increases [37,38]. This information is important because regardless of the component (ie, general use of Calm, specifically meditation, and specifically Sleep Stories), participants who used the app more frequently clearly noticed changes in their health. It is well known that individuals are more likely to participate in a behavior if they enjoy it or if they believe it benefits them [39,40]. However, it is important to note that survey questions about changes in all aspects of health were nondirectional, and respondents’ reports of noticing changes were interpreted with the assumption that changes reflect improvements. In addition, future research should explore the correlation between self-reported *noticing changes* and directional changes on validated outcome measures. It is possible that, retrospectively, users believe that there have been changes, but when completing a fully validated measure, scores remain the same over time. The relationship between actual and perceived outcomes and time spent (duration in minutes and length of membership) using the app would also be worth exploring in future research. Future research should also explore the optimal dose of mindfulness meditation on a mobile app to improve health outcomes. For example, it is possible that total time spent using a meditation app is less important than frequency of use it (eg, 10 min a day for 3 days could be better than 50 min in 1 day).

Respondents who used the Sleep Stories more frequently were more likely to report noticing changes in their sleep and physical health. Consumer sleep technologies have become quite popular to help individuals improve or self-monitor their sleep [20]. In a review by Ko in 2015 on consumer sleep technologies, a number of the top-rated sleep apps helped users track their *sleep trends* (movements that represent presence or absence of sleep), recorded ambient sounds (sleep talking and snoring), alerted users when to go to bed, and tracked their sleep habits over time. Although some of the apps were developed to facilitate sleep onset using visual graphics, relaxing music, and nature sounds, none incorporated Sleep Stories. Uniquely, Calm has Sleep Stories, similar to meditation, that help the user to practice moment-to-moment awareness, experience greater attentional control, and decrease ruminating thoughts, which may lead to less anxiety about sleeplessness [41,42]. The stories bring listeners focus into the present moment, helping them to disengage with negative thoughts/beliefs about sleeplessness, and provide cognitive distraction, which may lower presleep anxiety or arousal and help users sleep better.

It is important to note that those who used the Sleep Stories more frequently reported noticing changes in their sleep and physical health only, whereas those using meditations more frequently noticed changes in their mental and physical health, as well as stress. Although there is a large body of research demonstrating the relationship between sleep and physical health [43], many studies observe similar or stronger relationships between sleep and mental health [44,45]. Given this, it is surprising that using Sleep Stories was not associated with changes in mental health or stress. There may be more varying long-term benefits of using Calm for meditation as compared with sleep. Little research has been conducted to determine the effects of sleep components of mindfulness-based apps, and no studies have specifically assessed the effects of Calm Sleep Stories in improving sleep and health outcomes. Considering the increases in sleep components to mindfulness meditation apps, future research on the extent to which these components impact sleep is warranted.

### Limitations

There were a number of limitations, inherent in descriptive, survey-based studies. First, although this was a convenience sample of Calm users that opened an email from Calm and decided to complete the survey, engagement was still varied (60.03% of respondents used the app 5 or more times per week, 27.45% used 3-4 times per week, and 12.52% used only once or twice weekly), and there may be a viable sample of individuals who vary in their use of Calm. In addition, individuals who use Calm more frequently may have more favorable opinions of the app and have benefitted more from using it. Those who do not find Calm beneficial may have elected not to participate. In addition, the survey did not include the option to endorse using Calm less often than 1 to 2 times per week, which may have decreased participation rates of infrequent users. Finally, our sample is mostly white, female, and with higher income. Future studies should use stratified recruitment strategies to engage a more diverse sample in terms of age, gender, income, race/ethnicity, and pattern of usage. Second, as this was a cross-sectional, nonexperimental study, we cannot infer causality with regard to the impact of Calm; rather, analyses rely on subscribers’ recollections of app usage, perceptions of changes over time, and their beliefs about how Calm has impacted them. Future studies should explore these relationships prospectively, and randomized clinical trials are needed to determine extent to which changes can be attributed to Calm usage. Third, the survey items were designed specifically for this study, and thus, there is no previous research on their validity. In addition, questions asking participants to notice changes in their health, stress, and sleep after using Calm were dichotomous (yes/no), and results were interpreted with the assumption that changes reflect improvements. Future studies should extend the findings of this report by using validated questionnaires to assess directional changes in mental health, physical health, stress, and sleep outcomes. Finally, as many respondents used both meditations and Sleep Stories, it is difficult to disentangle the unique effects of those components. Future dismantling studies could randomize participants to only use 1 component of the app and look at the benefits of each component on its own. In addition, future surveys should collect more detailed information regarding the specific aspects of meditations and Sleep Stories that users enjoy, and this information should be presented alongside qualitative data, as such data could inform future experimental studies.

### Conclusions

This is the first study to report the demographic and clinical characteristics and patterns of usage among a large sample of paid subscribers to a top-rated health and fitness app, Calm. Results showed that most subscribers used meditation and Sleep Stories at least three to four times per week, and more frequent use was associated with perceived changes in their physical and mental health, stress, and sleep. We have also identified the most popular tools within the app preferred by subscribers. This paper provides information for Calm and other mindfulness-based apps about strategies to tailor content and features (eg, emphasize benefits for sleep, provide tracking with feedback, and incorporate social support that is private/anonymous) and potentially increase subscribers and retention rates. From a scientific perspective, these strategies can help participants engage in studies and facilitate feasibility. This study sets the stage for future clinical trials aimed at determining the efficacy of Calm in improving physical and mental health, stress, and sleep.

## Appendix

Multimedia Appendix 1 Screenshots of the home screen and meditation screen in Calm app.

Multimedia Appendix 2 Questions from Calm user engagement survey.

Multimedia Appendix 3 Additional details on statistical analyses of reasons for starting Calm.

Multimedia Appendix 4 Additional details on statistical analyses of Calm component usage.

Multimedia Appendix 5 Additional details on statistical analyses of changes in health stress and sleep after using Calm.

## Abbreviations

SAB: Scientific Advisory Board
