# Supplementary figures and images for: Characteristics and Usage Patterns Among 12,151 Paid Subscribers of the Calm Meditation App: Cross-Sectional Survey
Source: JMIR Mhealth Uhealth. 2019 Nov 3;7(11):e15648. doi: 10.2196/15648 (PMC6858610; doi:10.2196/15648)

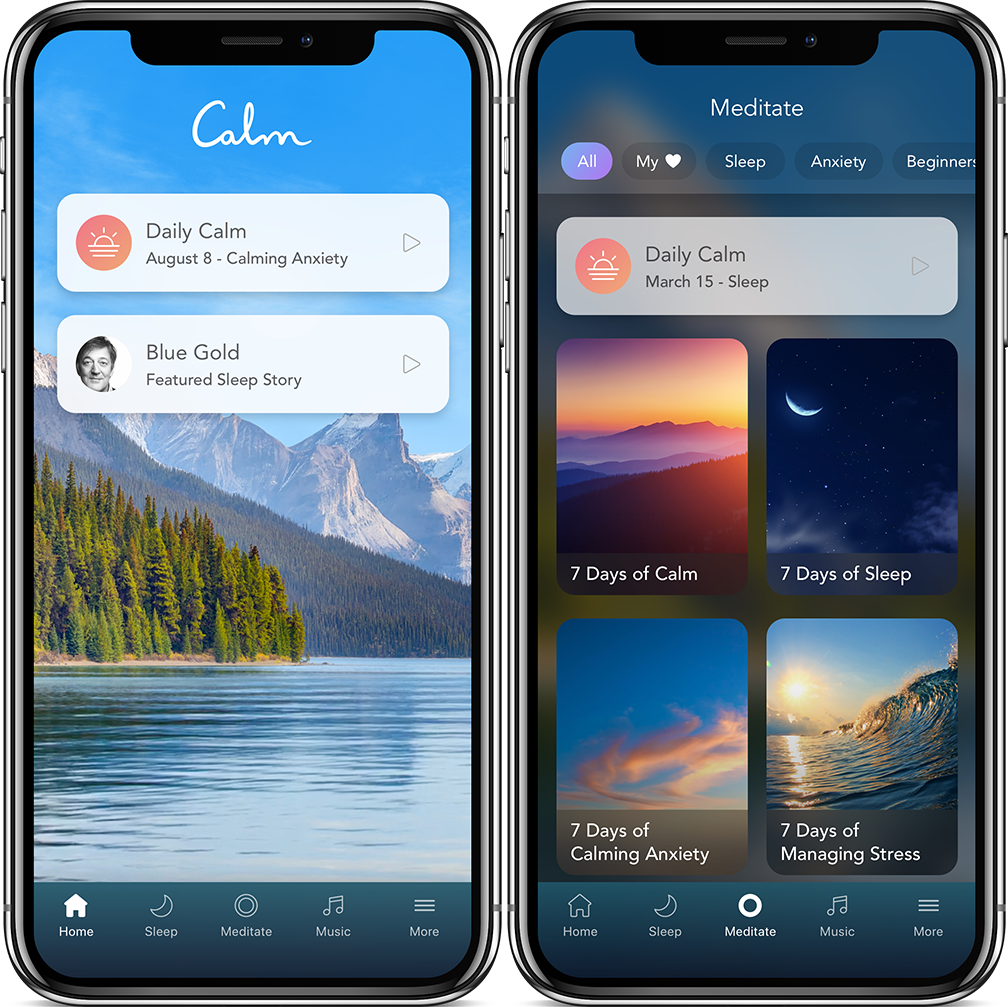

Supplement: Multimedia Appendix 1 [file mhealth_v7i11e15648_app1.png]
