# Supplementary material for: Characteristics and Usage Patterns Among 12,151 Paid Subscribers of the Calm Meditation App: Cross-Sectional Survey
Source: JMIR Mhealth Uhealth. 2019 Nov 3;7(11):e15648. doi: 10.2196/15648 (PMC6858610; doi:10.2196/15648)
Supplement: Multimedia Appendix 2 [file mhealth_v7i11e15648_app2.pdf]

Thank you for taking the time to complete this survey to help improve Calm. The results of this study may be used in reports, presentations, or publications. There are no foreseeable risks or discomforts to your participation.

As part of our research, we invite you to reflect on your usage of Calm and answer the questions below to help us to continue to improve your experiences! Please answer the questions to the best of your ability (there are no right or wrong answers). The survey will take less than 10 minutes of your time. If you are uncomfortable answering any of the questions, you have the right not to answer any question, and to stop participation at any time. Your responses will be anonymous and results will only be reported in the aggregate (collectively). You will, however, be given the opportunity at the end of the survey to provide your name and email address in order to be included in a drawing for one of two $99 gift cards to Amazon.

You must be 18 years or older to participate in the study.

If you have any questions concerning this survey, please contact Jennifer Huberty at science@calm.com. If you have any questions about your rights as a participant in this research, or if you feel you have been placed at risk, you can contact the Chair of the Human Subjects Institutional Review Board, through the ASU Office of Research Integrity and Assurance, at 480.965.6788.

Click 'continue' below if you consent to participate in the study.

--

1. How often do you use the Calm app?
   - o 1-2 times per week
   - o 3-5 times per week
   - o More than 5 times per week

2. Why did you start using the Calm app?
   - ✓ Improve overall health
   - ✓ Reduce stress
   - ✓ Reduce depression/anxiety
   - ✓ Improve sleep
   - ✓ Friend recommended the app
   - ✓ Someone bought it for me
   - ✓ Curious
   - ✓ Other (please describe):

3. When you downloaded Calm, had you been diagnosed with any of the following chronic conditions?
   - ✓ Anxiety
   - ✓ Post-Traumatic Stress Disorder
   - ✓ Depression
   - ✓ High blood pressure (Hypertension)
   - ✓ High cholesterol

- ✓ Diabetes
- ✓ Asthma
- ✓ Emphysema or COPD
- ✓ Other lung disease
- ✓ Heart disease
- ✓ Arthritis or other rheumatic disease
- ✓ Cancer
- ✓ Pain
- ✓ Insomnia
- ✓ Other chronic condition (please describe)
- ✓ None

4. Low-Frequency Users: Which component(s) of the app do you complete 1-2 times per week?
High-Frequency Users: Which component(s) of the app do you use most often?

- ✓ Meditation
- ✓ Sleep Stories
- ✓ Calm Body
- ✓ Breathe Bubble
- ✓ Masterclass
- ✓ Background sounds or scenes
- ✓ Music
- ✓ Other (please describe)

  High-Frequency Users:
  For selected Components: How often do you use [Component] on the Calm app?
    - o 1-2 times per week
    - o 3-4 times per week
    - o More than 5 times per week

5. When you first started using Calm, did you have difficulty falling asleep or staying asleep?

- o Falling asleep
- o Staying asleep
- o Both
- o Neither (no sleep difficulty)

6. Have you used any of the following to help increase how frequently you use the app?

- ✓ Share status
- ✓ Tracking of meditation sessions and minutes in each session
- ✓ Reminders
- ✓ Participation with the Calm Facebook community
- ✓ High-Frequency Users: I use the app for sleep before bed each evening
- ✓ High-Frequency Users: I am committed to using the app
- ✓ Other (please describe)
- ✓ Low-Frequency Users: None
  - o For selected Features: Did the [Feature] help you?
    - o Yes (How?)

        - No
    - If None: Why haven't you tried any of the features?
    - If None: What could we do to help you?

7: For how many months have you been using Calm?

8a. Have you noticed any changes in your physical health after using Calm?
8b. Have you noticed any changes in your mental health after using Calm?
8c. Have you noticed any changes in your sleep after using Calm?
8d. Have you noticed any changes in your stress level after using Calm?
    - Yes
    - No

9. Research shows that it is easier to remember to use an app if friends and family are also using the same app and can share progress with each other. Do any of your friends, and/or family members use Calm?
- Yes
- No

10. If friends and/or family members use Calm: Do you communicate with your friends/family about using the Calm app?
- Yes
- No

11. If friends and/or family members use Calm: Would you want to also be connected with other app users?
If no friends and/or family members use Calm Would you want to be connected with other app users?
- Yes
- No

12. What do you like most about the Calm app?

13. What do you like least about the Calm app?

14. High-Frequency Users: Is there anything that you would like to change about the app that would further contribute to your regular participation?

15. What year were you born?

16: How would you describe yourself? (Choose one or more from the following ethnic groups).
- American Indian or Alaskan Native
- Asian or Asian-American
- Native Hawaiian or Pacific Islander
- White, European-American, or Caucasian
- Black, African-American, or Native African
- Arab or Non-Arab North African/Middle-Eastern
- Native Caribbean or Afro-Caribbean Islander

- Bi-racial or Multi-racial
- Other (please specify)
- I prefer not to respond

17. What is your gender?
    - Female
    - Male
    - Transgender
    - Other (please describe)
    - Prefer not to say

18. What is your household income?
    - Less than $20,000 per year
    - $21,000-$40,000
    - $41-$60,000
    - $61,000-$80,000
    - $81,000-$100,000
    - $101,000-$150,000
    - $151,000-$200,000
    - $201,000-$250,000
    - $250,000 or more per year

--

If you would like to be included in a drawing for one of two $99 gift cards to Amazon, please include your email below.

Thank you for taking the time to complete this survey!
