# Supplementary material for: Characteristics and Usage Patterns Among 12,151 Paid Subscribers of the Calm Meditation App: Cross-Sectional Survey
Source: JMIR Mhealth Uhealth. 2019 Nov 3;7(11):e15648. doi: 10.2196/15648 (PMC6858610; doi:10.2196/15648)
Supplement: Multimedia Appendix 3 [file mhealth_v7i11e15648_app3.pdf]

Multimedia Appendix 3.1. Self-Reported Reasons for Starting to Use Calm (N = 11870)

Reason for starting Calm[a]	*n* (%)
Improve sleep	7475 (62.97%)
Improve stress	7373 (62.11%)
Reduce depression or anxiety	6465 (54.47%)
Improve overall health	4754 (40.05%)
Curious	1768 (14.89%)
Friend recommended the app	1717 (14.47%)
Someone bought it for me	197 (1.66%)
Other	1321 (11.13%)

[a]Participants were able to select multiple reasons for starting to use Calm

Multimedia Appendix 3.2. Differences in the Reasons for Starting Calm by Sleep Difficulties (N = 11380)

Reason for starting Calm[a]	Sleep difficulties (*N* = 8704), n (%)	No sleep difficulties (*N* = 2676), n (%)	X_1^2	*P*
Improve sleep	6597 (75.79)	579 (21.64)	2576.88	> .001
Improve stress	5381 (61.82)	1699 (63.49)	2.42	.12
Reduce depression or anxiety	4796 (55.10)	1404 (52.47)	5.73	.02
Improve overall health	3218 (36.97)	1331 (49.74)	139.03	> .001
Curious	1120 (12.87)	582 (21.75)	126.93	> .001
Friend recommended it app	12.38 (14.22)	409 (15.28)	1.86	.17
Someone bought it for me	140 (1.61)	45 (1.68)	.07	.79
Other	846 (9.72)	440 (16.44)	92.29	> .001

[a]Participants were able to select multiple reasons for starting to use Calm
