# Supplementary material for: Characteristics and Usage Patterns Among 12,151 Paid Subscribers of the Calm Meditation App: Cross-Sectional Survey
Source: JMIR Mhealth Uhealth. 2019 Nov 3;7(11):e15648. doi: 10.2196/15648 (PMC6858610; doi:10.2196/15648)
Supplement: Multimedia Appendix 4 [file mhealth_v7i11e15648_app4.pdf]

Multimedia Appendix 4.1. Use of Components of Calm App (N = 11841)

Component	Respondents regularly using component, *n* (%)
Meditations	9497 (80.02)
Sleep Stories	6591 (55.66)
Masterclass	1957 (16.53)
Calm Body	1689 (14.26)
Breathe Bubble	976 (8.24)
Music	2674 (22.01)
Background scenes	3483 (28.66)
Other	406 (3.34)

Multimedia Appendix 4.2. Sex Differences in the Components of Calm Regularly Used (N = 10955)

Component	Males (*N* = 2177), *n* (%)	Females (*N* = 8778), *n* (%)	χ_1^2	*P*
Meditations	1924 (88.38)	6835 (77.87)	120.30	< .001
Sleep Stories	771 (35.42)	5304 (60.42)	441.63	< .001
Masterclass	363 (16.67)	1451 (16.53)	0.03	.87
Calm Body	266 (12.22)	1294 (14.74)	9.09	.003
Breathe Bubble	140 (6.43)	748 (8.52)	10.23	.001
Music	468 (21.50)	1987 (22.64)	1.30	.25
Backgrounds	570 (26.18)	2653 (30.22)	13.71	< .001
Other	79 (3.63)	296 (3.37)	0.35	.56

Multimedia Appendix 4.3. Associations Between Age and the Components of Calm Regularly Used (N = 11014)

Component	B (Age)	SE	Wald	*df*	*P*	OR	95% CI
Meditations	-0.01	0.002	66.31	1	< .001	0.99	0.98 – 0.99
Sleep Stories	0.01	0.001	37.31	1	< .001	1.01	1.006 – 1.011
Masterclass	-0.004	0.002	3.88	1	.049	1.00	0.99 – 1.00
Calm Body	0.02	0.002	64.26	1	< .001	1.02	1.01 – 1.02
Breathe Bubble	-0.01	0.003	32.93	1	< .001	0.99	0.98 – 0.99
Music	-0.01	0.002	15.79	1	< .001	0.99	0.99 – 1.00
Backgrounds	-0.01	0.002	16.39	1	< .001	0.99	0.99 – 1.00
Other	0.02	.004	17.65	1	< .001	1.02	1.01 – 1.02

Multimedia Appendix 4.4. Differences in the Components of Calm Regularly Used by Types of Health Diagnoses (N = 11061)

Component	MH only (*N* = 2729), n (%)	PH only (*N* = 1740), n (%)	MH and PH (*N* = 1820), n (%)	None (*N* = 4772), n (%)	X_3^2	*P*
Meditation	2279 (83.51)	1325 (76.15)	1434 (78.79)	3808 (79.80)	38.88	< .001
Sleep Stories	1515 (55.51)	1021 (58.68)	1116 (61.32)	2495 (52.28)	52.04	< .001
Masterclass	432 (15.83)	258 (14.83)	307 (16.87)	835 (17.50)	7.99	.046
Calm Body	370 (13.56)	269 (15.46)	355 (19.51)	585 (12.26)	59.68	< .001
Breathe Bubble	265 (9.71)	128 (7.36)	175 (9.62)	342 (7.17)	21.45	< .001
Music	652 (23.89)	371 (21.32)	463 (25.44)	1021 (21.40)	16.51	.001
Background scenes	842 (30.85)	517 (29.71)	651 (35.77)	1270 (26.61)	55.66	< .001
Other	68 (2.49)	74 (4.25%)	75 (4.12)	158 (3.31)	13.74	.003

Multimedia Appendix 4.5. Pairwise Comparisons of the Components of Calm Regularly Used by Types of Health Diagnoses (N = 11061)

Component	MH only (A)	PH only (B)	MH and PH (C)	None (D)
Meditation	B, *P* < .001<br>C, *P* < .001<br>D, *P* < .001			B, *P* = .01
Sleep Stories	D, *P* = .04	D, *P* < .001	A, *P* = .001<br>D, *P* < .001	
Masterclass				
Calm Body		D, *P* = .004	A, *P* < .001<br>B, *P* = .01<br>D, *P* < .001	
Breathe Bubble	B, *P* = .04<br>D, *P* = .001		D, *P* = .01	
Music			B, *P* = .02<br>D, *P* = .003	
Background scenes	D, *P* = .001		A, *P* = .003<br>B, *P* = .001<br>D, *P* < .001	
Other		A, *P* = .01	A, *P* = .01	

Results are based on two-sided tests. For each significant pair, the key of the category with the smaller column proportion appears in the category with the larger column proportion.
Significance level for upper case letters (A, B, C, D) = .05.
Tests are adjusted for all pairwise comparisons within a row of each innermost subtable using the Bonferroni correction.

Multimedia Appendix 4.6. Differences in the Components of Calm Regularly Used by Sleep Difficulties (N = 11380)

Component	Sleep difficulties (*N* = 8527), n (%)	No sleep difficulties (*N* = 2604), n (%)	X_1^2	*P*
Meditation	6677 (76.71)	2431 (90.84)	255.84	< .001
Sleep Stories	5589 (64.21)	755 (28.21)	1075.12	< .001
Masterclass	1374 (15.79)	497 (18.57)	11.57	.001
Calm Body	1257 (14.44)	365 (13.64)	1.08	.30
Breathe Bubble	699 (8.03)	244 (9.12)	3.18	.07
Music	1980 (22.75)	584 (21.84)	1.00	.32
Background scenes	2570 (29.53)	770 (28.77)	0.56	.46
Other	296 (3.40)	93 (3.48)	.04	.85
