# Supplementary material for: Characteristics and Usage Patterns Among 12,151 Paid Subscribers of the Calm Meditation App: Cross-Sectional Survey
Source: JMIR Mhealth Uhealth. 2019 Nov 3;7(11):e15648. doi: 10.2196/15648 (PMC6858610; doi:10.2196/15648)
Supplement: Multimedia Appendix 5 [file mhealth_v7i11e15648_app5.pdf]

Multimedia Appendix 5.1 Differences in Noticing Changes in Mental Health (N = 11170), Physical Health (N = 11161), Stress (N = 11123), and Sleep (N = 11189) Based on Frequency of Using of Calm Components

Component and reported changes after using	< 1 time, *n* (%)	1-2 times, *n* (%)	3-4 times, *n* (%)	≥ 5 times, *n* (%)	X_3^2	*P*
Meditations						
Mental health	1209 (54.56)	1490 (78.46)	2430 (85.96)	3862 (91.34)	1325.02	< .001
Physical health	1053 (47.14)	973 (51.51)	1598 (56.47)	2573 (61.15)	130.86	< .001
Stress	1192 (53.94)	1428 (75.76)	2386 (84.61)	3718 (88.36)	1100.47	< .001
Sleep	1890 (84.07)	1304 (69.03)	1812 (63.98)	2778 (65.83)	293.30	< .001
Sleep Stories						
Mental health	4272 (85.78)	1696 (80.46)	1381 (74.69)	1642 (73.53)	197.31	< .001
Physical health	2721 (54.84)	1113 (52.92)	1016 (54.65)	1347 (60.21)	27.21	< .001
Stress	4181 (84.24)	1607 (77.00)	1341 (72.53)	1595 (71.72)	199.01	< .001
Sleep	2551 (50.84)	1545 (73.47)	1641 (87.94)	2077 (91.86)	1665.31	< .001
Masterclass						
Mental health	7390 (78.70)	1425 (90.13)	149 (89.22)	27 (84.38)	121.18	< .001
Physical health	5044 (53.79)	1019 (64.41)	110 (65.09)	24 (72.73)	72.23	< .001
Stress	7143 (76.44)	1403 (88.85)	153 (91.07)	25 (80.65)	139.33	< .001
Sleep	6547 (69.59)	1088 (68.86)	121 (72.02)	28 (84.85)	4.94	.213
Calm Body						
Mental health	7640 (76.53)	823 (85.64)	384 (86.69)	144 (90.00)	41.86	< .001
Physical health	5140 (53.56)	621 (64.62)	316 (71.33)	120 (75.00)	116.62	< .001
Stress	7383 (77.21)	812 (84.67)	393 (88.51)	136 (86.08)	62.62	< .001
Sleep	6660 (69.22)	660 (68.61)	347 (77.80)	117 (73.13)	16.20	.001
Breathe Bubble						
Mental health	8184 (79.77)	585 (88.64)	166 (88.30)	56 (90.32)	42.44	< .001
Physical health	5626 (54.88)	405 (61.27)	124 (66.67)	42 (67.74)	26.68	< .001
Stress	7928 (77.60)	581 (88.03)	160 (86.02)	55 (91.67)	52.71	< .001
Sleep	7162 (69.66)	436 (66.46)	136 (71.96)	50 (80.65)	7.13	.07
Music						
Mental health	6957 (80.29)	965 (82.98)	635 (77.34)	434 (83.30)	12.59	.01
Physical health	4700 (54.34)	675 (57.94)	483 (58.69)	339 (64.82)	29.33	< .001
Stress	6683 (77.43)	964 (83.32)	653 (79.73)	424 (82.17)	26.54	< .001
Sleep	5955 (68.57)	803 (69.22)	611 (74.15)	415 (79.65)	37.33	< .001
Backgrounds						
Mental health	6199 (78.51)	850 (81.34)	751 (83.35)	1191 (89.68)	96.42	< .001
Physical health	4185 (53.00)	569 (84.61)	573 (63.81)	870 (65.66)	100.79	< .001
Stress	5981 (76.10)	835 (80.06)	751 (83.44)	1157 (87.59)	105.60	< .001
Sleep	5406 (68.32)	691 (66.38)	687 (75.74)	1000 (75.30)	47.80	< .001
Other						
Mental health	8687 (80.34)	101 (81.45)	85 (83.33)	129 (88.97)	7.34	.06
Physical health	5968 (55.30)	73 (59.84)	59 (57.84)	97 (67.36)	9.54	.02
Stress	8426 (78.31)	96 (79.34)	79 (78.22)	123 (87.23)	6.62	.09
Sleep	7527 (69.57)	80 (64.52)	72 (71.29)	105 (72.92)	2.40	.49

Multimedia Appendix 5.2. Pairwise Comparisons of Noticing Changes in Mental Health, Physical Health, Stress, and Sleep Based on Frequency of Using of Calm Components

Component and reported changes after using	< 1 time (A)	1-2 times (B)	3-4 times (C)	≥ 5 times (D)
Meditations				
Mental health		A, $P < .001$	A, $P < .001$<br>B, $P = .01$	A, $P < .001$<br>B, $P < .001$<br>C, $P = .001$
Physical health		A, $P = .03$	A, $P < .001$<br>B, $P = .01$	A, $P < .001$<br>B, $P < .001$<br>C, $P = .001$
Stress		A, $P < .001$	A, $P < .001$<br>B, $P < .001$	A, $P < .001$<br>B, $P < .001$<br>C, $P < .001$
Sleep	B, $P < .001$<br>C, $P < .001$<br>D, $P < .001$	C, $P = .002$		
Sleep Stories				
Mental health	B, $P < .001$<br>C, $P < .001$<br>D, $P < .001$	C, $P < .001$<br>D, $P < .001$		
Physical health				A, $P < .001$<br>B, $P < .001$<br>C, $P = .002$
Stress	B, $P < .001$<br>C, $P < .001$<br>D, $P < .001$	C, $P = .01$<br>D, $P < .001$		
Sleep		A, $P < .001$	A, $P < .001$<br>B, $P < .001$	A, $P < .001$<br>B, $P < .001$<br>C, $P < .001$
Masterclass				
Mental health		A, $P < .001$	A, $P = .01$	
Physical health		A, $P < .001$	A, $P = .02$	
Stress		A, $P < .001$	A, $P < .001$	
Sleep				
Calm Body				
Mental health		A, $P < .001$	A, $P = .001$	A, $P = .01$
Physical health		A, $P < .001$	A, $P < .001$	A, $P < .001$
Stress		A, $P < .001$	A, $P < .001$	A, $P = .050$

Sleep		A, $P = .001$<br>B, $P = .002$	
Breathe Bubble			
Mental health	A, $P < .001$	A, $P = .02$	
Physical health	A, $P = .01$	A, $P = .01$	
Stress	A, $P < .001$	A, $P = .04$	
Sleep			
Music			
Mental health	C, $P = .01$		C, $P = .049$
Physical health			A, $P < .001$<br>B, $P = .046$
Stress	A, $P < .001$		
Sleep		A, $P = .01$	A, $P < .001$<br>B, $P < .001$
Background scenes			
Mental health		A, $P = .004$	A, $P < .001$<br>B, $P < .001$<br>C, $P < .001$
Physical health		A, $P < .001$<br>B, $P < .001$	A, $P < .001$<br>B, $P < .001$
Stress	A, $P = .03$	A, $P < .001$	A, $P < .001$<br>B, $P < .001$<br>C, $P = .04$
Sleep		A, $P < .001$<br>B, $P < .001$	A, $P < .001$<br>B, $P < .001$
Other			
Mental health			
Physical health			A, $P = .02$
Stress			
Sleep			

Note. For each significant pair, the key of the category with the smaller column proportion appears in the category with the larger column proportion. Significance level for upper case letters (A, B, C, D): 05. Tests are adjusted for all pairwise comparisons within a row using the Bonferroni correction.
